# Intraoral repair of fractured ceramics: a literature review

**DOI:** 10.12688/f1000research.126547.1

**Published:** 2022-11-08

**Authors:** Firas K. Alqarawi, Abdulkareem A. Alhumaidan, Abdulrahman Aldakhili, Abdullah Alfayez, Tahani Abushowmi, Anwar Alramadan, Fawaz Alzoubi

**Affiliations:** 1 Department of Substitutive Dental Sciences, College of Dentistry, Imam Abdulrahman Bin Faisal University, Dammam, Eastern Region, 31441/P.O. Box 1982, Saudi Arabia; 2 Department of Preventive Dental Sciences, College of Dentistry, Imam Abdulrahman Bin Faisal University, Dammam, Eastern Region, 31441/P.O. Box 1982, Saudi Arabia; 3 Department of General Dental Practice, Faculty of Dentistry, Kuwait University, Safat, 13110/P.O. Box 24923, Kuwait

**Keywords:** Intraoral, restoration, ceramics, fixed partial dentures, zirconia, porcelain chipping, veneering.

## Abstract

**Background:** Fracture or chipping of veneering ceramic resulting in aesthetic and functional issues is a frequent technical complication encountered with fixed dental prostheses. Treatment options include extraoral or intraoral repair of the ceramic restoration; the latter being minimally invasive and cost-effective. We reviewed the various intraoral repair techniques for ceramic fractures and their efficiency in the last decade.

**Methods:** A literature search was carried out between 2017 and 2022 using the PubMed database with keywords: intraoral, repair, ceramics, porcelain, and ‘porcelain fused to metal’. Screening of abstracts and full texts was carried out to determine the final list of eligible studies.

**Results:**  Twenty-one eligible studies were included in the review which consisted of 17 in vitro studies, three case reports, and one prospective clinical study. Researchers and dentists preferred repairing cracked veneered zirconia-based restorations intraorally if the restoration is in acceptable condition. However, successful intraoral repair of veneering fractures relies on the bond between the previous restoration and the new repair material, the right adhesive resin, and adequate surface conditioning. The review indicated that the best technique to fix chipping in a complex made of zirconia core and veneering ceramic is to treat the veneering ceramic with hydrofluoric acid before covering the core ceramic with silica.

**Conclusion:** Intraoral repair is an efficient and economic procedure without the need for repeated sessions. However, the success and longevity of the restoration rely on the technical skills of the clinicians and adherence to prescribed protocols.

## 1. Introduction

Modern cosmetic dentistry places a strong emphasis on aesthetics, and thus finds a wide usage of ceramic and porcelain in teeth restoration to achieve the most natural-looking aesthetics. However, the accelerated usage of dental porcelains is accompanied by the risk of fractures ( Hammond *et al.* 2009). Owing to its brittle nature, porcelain veneering, when exposed to masticatory forces, clenching, and moisture causes cracks or chipping ( Rosentritt, Steiger *et al.* 2009). Studies have shown that the most frequent technical issue associated with dental restorations were ceramic fractures, which were followed by porcelain chipping and loss of retention ( Vagropoulou, Klifopoulou *et al.* 2018). According to a systemic literature review *,* chipping of the veneering ceramic is one of the most frequent technical complications occurring at a rate of 12.7% following a three-year observation period ( Pjetursson, Sailer *et al.* 2015). Consequently, patients with ceramic fractures might have aesthetic and functional issues that need an immediate fix. Direct repairs have been suggested in such conditions where the broken prosthesis displays good adaptation and acceptable aesthetics ( Mesquita, Al-Haj Husain *et al.* 2021).

In bilayered dental prostheses, such as metal-ceramic fixed dental prostheses (FDP), fractures in veneering ceramic may be treated either by replacing the existing restoration or by repairing it intraorally. Removal of FDP can be challenging as it can lead to deformation of the metal and iatrogenic fracture of the sound tooth tissues that can seriously compromise the longevity of the tooth. Additionally, replacing FDPs is typically a difficult and expensive process that adds significantly to chairside time, which is not always well received by the patient ( Özcan and Volpato 2017). Furthermore, it is associated with the risk of harming the tooth that serves as the abutment ( Kocaağaoğlu, Manav *et al.* 2017).

On the other hand, intra-oral ceramic restoration repair is minimally invasive and most cost-effective making it an ideal choice ( Aslam, Hassan *et al.* 2018). Furthermore, intraoral repair has recently been suggested as a potential therapy alternative if the indications and treatment techniques are suitable ( Agingu, Zhang *et al.* 2018). Repairing the ceramic restoration intraorally is a temporary but effective solution. The easiest way is to polish the surface and prevent future failures. If the repair cannot be done by polishing, the missing part is replaced with composite resin, or the chipped part is cemented using resin cement. A new veneer layer is prepared and bonded to the existing restoration ( Kimmich and Stappert 2013).

Intraoral repair is a simpler technique to treat veneer ceramic fractures than total replacement or extraoral repair methods ( Polat, Tokar *et al.* 2021). Veneering porcelain flaws that expose the restoration's core material create a special challenge for intraoral repair. Particularly with metal frameworks, it can be challenging for the dentist to match the colour of the framework with veneer without compromising the aesthetic outcome. To achieve functional success, the dentist must effectively bond the veneering porcelain to the core material ( Kimmich and Stappert 2013). However, selecting the best system that would deliver a trustworthy result is often difficult for clinicians. Hence, this article reviews studies that have investigated intraoral repair techniques for ceramic fractures and their efficiency in the last five years.

## 2. Methods

### 2.1 Data sources and search strategy

The literature search was conducted using the PubMed database for studies that investigated intraoral repair techniques for ceramic fractures. All studies published in English from January 2017 through August 2022 were considered for review. The search strategy was derived from appropriate keywords representing the concepts: “intraoral repair”, “ceramics”, “porcelain”, “porcelain fused to metal”, and “efficacy”. The primary outcome of the review included the efficacy of the intraoral repair technique used. Articles were excluded if published in a language other than English, not published in the last five years, or if full text is unavailable.

After applying the criteria, study selection was undertaken by first screening the abstract and title followed by the full-text versions of relevant studies to determine the final list of eligible studies. Data were extracted from the studies following the completion of full-text screening. All data were summarized. No statistical analysis was performed.

## 3. Results

The literature search from PubMed retrieved 21 eligible studies that were included in this review to understand the intraoral repair techniques used for treating ceramic fractures. The findings of the included studies have been summarized in Table 1.

**Table 1.** Literature overview of intraoral repair systems for fractured ceramic restorations.

Sl. No.	Author, year	Framework material/Type of ceramic	Manufacturer	Surface treatment	Repair system	Study design	Number of participants/samples	Age	Conclusion
1	Kiomarsi *et al.*, 2022	Lucite-reinforced glass-ceramic plates	IPS Empress; IvoclarVivadent, Liechtenstein	Laser and universal adhesive	Composite resin	*In vitro*	80	NA	In comparison to HF plus Universal adhesive, ceramic surface treatment with HF plus silane plus traditional adhesive produced a greater SBS.
2	Höller *et al.*, 2022	Lithium disilicate glass ceramic	Ivoclar Vivadent; Schaan, Liechtenstein	1. Self-etching glass-ceramic primer (MEP) 2. Grit blasting	NR	*In vitro*	60	NA	Intraoral repair of lithium disilicate glass ceramic when utilising GBL, should include the usage of rubber dam
3	Benli *et al.*, 2022	Zirconia-Reinforced Lithium Silicate Ceramics	Celtra Duo, Dentsply De Trey, Bensheim, Germany	1. Group H = HF etching 2. Group N = Nd: YAG (neodymium: yttrium-aluminum-garnet) laser 3. Group NH = Nd: YAG laser + HF gel 4. Group E = Er: YAG (erbium: yttrium-aluminum-garnet) laser 5. Group EH = Er: YAG laser + HF gel	NR	*In vitro*	100	NA	Clinicians may favour Nd: YAG laser therapy or Nd: YAG laser therapy combined with HF to intraorally repair fractured areas
4	Polat *et al.*, 2021	1. 100% Zirconia	1. ICE Zirkon Translucent	1. Control: Grinding	Clearfil Repair and Clearfil Majesty Esthetic	*In vitro*	120	NA	a. For each level of cracked zirconia ceramic, griding with a diamond bur improved the bond strength needed for repair
		2. 100% Veneering ceramic	2. Vita VMK Master	2. Sandblasting					b. To get an appropriate binding strength between composite resin and a broken zirconia framework, the manufacturer's instructions must be followed
		3. 50% Veneering ceramic + 50% Zirconia		3. Long pulse laser					
				4. Short pulse laser					
5	Mesquita *et al.*, 2021	1. Zirconia framework	1. Lava	Air abrasion, Etchant, Silane	Clearfil Photoposterior	Case Report	1	-	When using a direct repair procedure, a problem resulting from a veneering ceramic's fracture or chipping can be resolved promptly and effectively without the need to replace the prosthesis
		2. Veneering ceramic	2. IPS e.max Ceram						
6	Hakimaneh *et al.*, 2020	Feldspathic porcelain	Kuraray Noritake Dental Inc	1. Etchant+Silane	Tetric N-Ceram	*In vitro*	60	NA	To strengthen the link between porcelain and composite resin, Er: YAG irradiation prior to silane treatment is just as advantageous as hydrofluoric (HF) acid etching and silane treatment
				2. Silane+CO2 laser					
				3. CO2 laser+Silane					
				4. Silane+Er: YAG laser					
				5. Er: YAG laser+Silane					
				6. Bur+Etchant+Silane					
7	Meirelles *et al.*, 2020	Porcelain veneered zirconia FPD	VITA Zahnfabrik	Etchant and Silane	FILTEK Z350	Case Report	1	48 years	Intraoral repairs of chipped restoration were done in single sitting using composite resin that survived for more than 5 years
8	Sanal and Kilinc, 2020	Ceramic specimen (VITA VM 9; VITA VM 13; VITA VMK 95; and IPS e.max Ceram)	VITA Zahnfabrik	Air abrasion	1. Vertise flow	*In vitro*	96	NA	Self-adhesive composites could be used instead of a repair kit with flowable composite for repairing chipping fractures of ceramics
					2. Fusio Liquid Dentin				
					3. Constic				
					4. BISCO + Filtek Supreme				
9	Gul and Altınok-Uygun, 2020	One feldspar ceramic and two resin nanoceramics	Vitablocks Mark II	1. Control Group	Composite resin	*In vitro*	96	NA	a. The ceramic type determines the success of CAD/CAM blocks
			Lava Ultimate and Cerasmart	2. Acid Etching					b. Resin nanoceramics are more successful in intraoral repair applications
				3. CoJet System					
				4. Z-Prime Plus					
				5. GC Repair					
				6. Cimara System					
				7. Porcelain Repair System					
				8. Clearfil repair system					
10	Dos Santos *et al.*, 2019	Zirconia ceramic	IPS e. max ZirCAD, Ivoclar-Vivadent	1. Polished zirconia All Bond Universal 2. Polished zirconia Single Bond Universal 3. Polished zirconia (control) Z-Prime Plus 4. Polished zirconia Z-Prime Plus + All Bond Universal 5. Grit-blasted zirconia Single Bond Universal 6. Grit-blasted zirconia All Bond Universal 7. Grit-blasted zirconia (control) Z-Prime Plus 8.Grit-blasted zirconia Z-Prime Plus + All Bond Universal	Scotch bond Universal (3M ESPE) All Bond Universal (Bisco) Z-Prime Plus (Bisco) zirconia primer	*In vitro*	80	NA	Universal adhesive systems make zirconia bonding easier and make intraoral repair of fracture possible
11	Gerogianni *et al.*, 2019	Lithium disilicate	IPS e.max Press and IPS e.max CAD	NR	Fabrication technique	*In vitro*	60	NA	In comparison to the milling, the pressed fabrication method produced lithium disilicate restorations with much higher crown strength and fracture resistance
12	Passia *et al.*, 2019	Veneered yttrium-oxide partially stabilized zirconia ceramic	CerconBase30, Degudent CerconCeramS, Degudent	NR	Fixed-to-fixed design, cantilever design	Prospective clinical study	48	Mean age of women 55.7 years mean age of men 54.3 years	After 13 years of clinical monitoring, posterior FDP composed of veneered zirconia with either a fixed-to-fixed or a cantilever design exhibit equivalent survival and success rates
13	Yadav *et al.*, 2019	Nickel chromium base metal alloy with ceramic layer	1. Bella bond plus (base metal)	Etchant and Primer	Clearfil porcelain repair system, Shofu (P and R) repair system	*In vitro*	90	NA	a. Cohesive fractures are easier to repair than adhesive fractures
			2. Ceramco 3 (ceramic)						b. The Clearfil repair material outperforms the P and R material utilized in this investigation for cohesive fractures
									c. For adhesive fractures, the material of choice is P and R repair material
14	Garbelotto *et al.*, 2019	Lithium disilicate based glass ceramic with feldspathic porcelain veneer	NR	Etchant and Silane	Estelite Sigma Quick composite repair system	Retrospective case report	1	60	a. Failure is a consequence of crack formation during function
									b. Repair using resin composite prolongs esthetics and function
15	Tokar *et al.*, 2019	Zirconia ceramic	ICE Zirkon Translucent	1. Control: Griding	Ceramic Repair N	*In vitro*	60	NA	a. The bond strength between zirconia and composite resin can be increased by applying Er, Cr: YSGG laser
				2. Short pulse laser					b. This laser irradiation can be safely used as a chairside treatment method to repair fractured ceramic restorations
				3. Long pulse laser					
16	Tatar and Ural, 2018	1. Polymer infiltrated ceramic	1. Vita Enamic	1. Control group	Filtek Z250	*In vitro*	48	NA	a. Different polishing properties and chemical structures directly affect the surface properties and bond strength
				2. Etchant					
		2. Resin nano ceramic	2. Lava Ultimate	3. Etchant+Silane coupling agent					
				4. Sandblasting+Silane coupling agent					
17	Libecki *et al.*, 2017	Zirconia ceramic	Cercon, DeguDent	1. Silicon carbide paper	Bifix QM	*In vitro*	48	NA	a. Surface conditioning with air abrasion and roughening of zirconia showed durable tensile bond strength after repair of zirconia ceramic due to chipping of veneers
				2. Air abrasion					
				3. Special silicon carbide grinder					
18	Kocaağaoğlu *et al.*, 2017	1. Zirconia	1. Zirkonzahn	Etchant and Silane	1. BISCO Intraoral repair kit	*In vitro*	90	NA	Ceramic repair systems can be used as productive and economical temporary treatment of fractured all ceramic restoration
		2. Alumina	2. In-Ceram Alumina, Vita Zahnfabrik		2. Cimara and Cimara Zircon Repair System				
		3. Glass ceramic	3. Vitablocks for CEREC		3. Clearfil Repair System				
19	Ozdemir and Yanikoglu, 2017	1. Vita porcelain	1. VMK95	1. Etchant+Silane	Nh.com and Nc.com resins	*In vitro*	120	NA	a. The shear bond strength (SBS) of composite resin to porcelain was affected differently by various surface treatments
		2. Ivoclar porcelain	2. IPS Classic	2. Air abrasion+Silane					b. The bonding of feldspathic porcelain is not improved by ceramic particles in the composite resin
				3. Air abrasion					
20	Sattabanasuk *et al.*, 2017	Leucite reinforced glass ceramic	IPS Empress Esthetic E O _2_	Etchant and Silane	Filtek Z350XT	*In vitro*	80	NA	a. The resin-ceramic bond depends on both mechanical and chemical retention
									b. For resin adherence to glass ceramic surfaces, a silane solution must always be utilized
21	Chen *et al.*, 2017	Human dentin	NA	NR	Composite resin	*In vitro*	-	-	a. Silane has no negative effects on dentin bond strength. cross contamination

The current literature review included 17 *in vitro* studies, three case reports, and one prospective clinical study. Various ceramic types were included in these studies such as glass-based ceramics with three subclasses: predominantly glass (feldspathic glass), moderately filled glass (leucite), and highly filled glass (leucite disilicate based); glass-infiltrated ceramics (In-Ceram groups); and non-glass-based ceramics: polycrystalline ceramics (alumina, zirconia). Likewise, various surface treatments such as polished surface, air abraded, or ground using a special silicon carbide bur (SiC Grinding Bur), yttrium-aluminum-garnet laser (Neodymium, Erbium, Chromium, Scandium, Gallium, CO _2_ laser) treatment were employed across the studies to improve the to increase the surface roughness and bond strength. The repair systems included in the articles were Clearfil Repair, Clearfil Majesty Esthetic, Clearfil Photoposterior, Clearfil porcelain repair system, Shofu (P and R) repair system, Estelite Sigma Quick composite repair system, Cimara, and Cimara Zircon Repair System and BISCO Intraoral repair kit.

The results of the studies included in this review are broadly divided into surface treatment methods and intraoral repair techniques employed in the studies.

### 3.1 Surface Treatment methods employed in the included studies

Most of the included studies have emphasized different surface treatment methods. An *in vitro* study from Iran compared the effectiveness of new ceramic surface treatments (laser and universal adhesive) with the traditional method for improving the bond strength of composite resin to ceramic during repairs. The group consisting of hydrofluoric (HF) acid, silane, and conventional adhesives had the highest shear bond strength (SBS) (mean: 12.0481 MPa) compared to the traditional adhesive and laser technology group (mean: 2.5766 MPa). HF acid surface treatment produced significantly greater SBS than laser (P < 0.001) ( Benli, Kilic *et al.* 2022, Kiomarsi, Jarrah *et al.* 2022). Holler *et al.* compared two distinct surface pretreatment techniques to repair a lithium-disilicate glass-ceramic (LDS) to see how they would react to simulated intraoral circumstances (increased temperature and humidity). Significantly less tensile bond strength (TBS) was produced by higher temperatures and humidity. Self-etching glass-ceramic primer (MEP), however, was a good option for intraoral ceramic repair since it was less susceptible to environmental factors than grit blasting (GBL). The results indicated that a rubber dam should be used during the clinical intraoral repair of lithium-disilicate glass ceramics, especially when utilising GBL ( Höller, Belli *et al.* 2022).

Santos *et al.* evaluated the bond strength of universal adhesive systems in comparison to a zirconia primer; and the bonding effectiveness of these materials onto different zirconia surface treatments. Grit-blasted zirconia promoted interlocking of universal adhesive systems to zirconia and had higher bond strength than polished ones ( Dos Santos, de Lima *et al.* 2019).

Benli *et al.* assessed the effect of two different lasers in combination with HF etching on the surface roughness of zirconia-reinforced lithium silicate (ZLS) ceramics and their SBS to composite resin. The mean SBS values for the HF etching group were highest for neodymium-doped yttrium aluminum garnet (Nd: YAG) laser (17.1 ± 1.65 MPa) and Nd: YAG laser + HF gel group (16.65 ± 1.11 MPa) making them the therapy of choice for the intraoral repair of fractured ceramics ( Benli, Kilic *et al.* 2022). Likewise, Hakimaneh *et al.* found heat treatment of silane with erbium-doped yttrium aluminum garnet (Er: YAG) and CO _2_ lasers to be effective as HF etching for treating fractured porcelain. However, this treatment failed to improve the micro SBS between feldspathic porcelain and composite resin ( Hakimaneh, Shayegh *et al.* 2020). Tokar *et al.* found that erbium, chromium: yttrium scandium gallium garnet (Er, Cr: YSGG) laser surface treatments with different pulses can be safely recommended in the treatment of fractured zirconia ceramics and can strengthen the SBS between zirconia and composite resin. Shorter pulse laser irradiation performed better compared to longer pulse rates in terms of mean SBS ( Tokar, Polat *et al.* 2019).

Sattabanasuk *et al.* assessed the efficacy of resin adhesion to glass-ceramic surfaces that had previously undergone various treatments and found that silane solution could be used to achieve mechanical and/or chemical retention. The study found that regardless of the tested adhesive, the resin-ceramic bond depended upon both mechanical and chemical retention. The authors suggested that adding a silane monomer to the adhesive formulation may not be the best way to achieve silanization and hence recommend regularly applying a silane solution to a glass-ceramic surface to ensure resin adherence ( Sattabanasuk, Charnchairerk *et al.* 2017).

Polat *et al.* compared the effectiveness of Er, Cr: YSGG laser with short and long pulse durations to different surface roughening techniques for repairing zirconia ceramics with various surface configurations and found there were no discernible variations among sandblasting and Er, Cr: YSGG laser treatments ( Polat, Tokar *et al.* 2021). Tatar and Ural investigated surface treatments for chipping to reduce the problems related to bonding between hybrid materials and composites and found that the most effective method was air abrasion with silica-coated aluminum oxide ( Tatar and Ural 2018). A similar study by Libecki *et al.* where the aim was to check the efficacy of different surface treatment options (air abraded, polished surface, or ground using a special silicon carbide bur), reported that the TBS is high when the ceramic is conditioned with air abrasion or roughened with silicon carbide bur ( Libecki, Elsayed *et al.* 2017). Ozdemir and Yanikoglu assessed the SBS of nano-hybrid and nano-ceramic composite resins to feldspathic porcelain (Vita and Ivoclar) and found the latter to possess lower SBS compared with the former ( Ozdemir and Yanikoglu 2017). A study by Chen *et al.* evaluated if silane contamination of dentin impacts the binding strength of the tissue and found that there were no adverse effects on SBS due to contamination of dentin with silane before etching ( Chen, Hammond *et al.* 2017).

### 3.2 Intraoral repair techniques in the included studies

Various intraoral techniques employed in the studies included in the review are listed below. Passia *et al.* evaluated the long-term outcome of posterior all-ceramic FDPs made from veneered zirconia ceramic with either a fixed-to-fixed (FF) or a cantilever design (CA). The cumulative 13-year survival rate was 43.2% (FF) (CI: 22.8–66.2%) and 52.5% (CA) (CI: 32.5–71.8); and success rate was 29.5% (FF) (CI:12.1–55.9%) and 22.5% (CA) (CI: 7.9–49.3%) respectively. The survival rates were comparable and failure rates were high, irrespective of the design ( Passia, Chaar *et al.* 2019). An in vitro study analyzed the long-term performance of fabrication techniques on anterior teeth and found that the pressed groups (923.7 N) exhibited significantly higher fracture resistance and crown strength than the milled groups (797.5 N), (p = 0.0002) ( Gerogianni, Lien *et al.* 2019). In a study by Sanal and Kilinc, SBS and color stability of four ceramic veneers (VITAVM 9, VITA VM 13, VITA VMK 95, and IPS e.max Ceram) treated with different repair systems (Vertise Flow, Fusio Liquid Dentin, Constic, and BISCO intraoral repair kit + Filtek Supreme) was examined. The findings of the study indicated that Constic self-adhesive composite resin had the lowest color stability value, but that there was no discernible difference between it and the other repair methods ( Sanal and Kilinc 2020).

A study by Meirelles *et al.,* reported five-year survival of intraoral repair of cracked porcelain veneered zirconia framework restoration with composite resin reconstruction, suggesting that it could be a suitable alternative therapy ( Meirelles, da Rocha *et al.* 2020). Gul and Uygun evaluated the micro-TBS of the nanocomposite resin to three CAD/CAM blocks utilizing various intraoral repair procedures. In comparison to the other repair methods examined, the Cimara System, Clearfil Repair, and Porcelain Repair systems significantly improved (p<0.05) the binding strength of nanohybrid resin composite to all CAD/CAM blocks ( Gul and Altınok-Uygun 2020). A case report by Mesquita *et al.* reported that proper execution of the direct veneering strategies yields fast and effective solutions to fracture or chipping of the veneering ceramic, without the need to replace the prosthesis. The durability of the repair relies on the quality of the interface created and the clinician's hold on the adhesive procedures ( Mesquita, Al-Haj Husain *et al.* 2021).

Yadav *et al.* reported that the Clearfil ^TM^ repair method had higher SBS (29.16 Mpa) than Primer and Cera resin bond repair material for cohesive fractures, whereas the latter had higher SBS (27.23 Mpa) for adhesive fractures compared to Clearfil ^TM^ repair method in metal-ceramic restorations ( Yadav, Dabas *et al.* 2019). The retrospective analysis conducted by Gabelotto *et al.* discussed the clinical phases of an intraoral composite resin repair and a fractographic examination of the failure reasons for the chipping of the veneering ceramic of an anterior single crown. Fractographic analysis revealed that failure was a result of crack development during function, most likely brought on by an occlusal interference, and thereby improved the knowledge of the origin of failure to avoid failures in the future ( Garbelotto, Fukushima *et al.* 2019).

## Discussion

The current review aimed to summarize the available literature on the effectiveness of intraoral repair techniques for ceramic fractures. Twenty-one relevant studies published in English between 2016 and 2022 which discussed various surface treatments and intraoral repair systems were included in the review.

It is a common observation in a dental practice that despite having a high success rate, chipping or fracture of the veneering ceramic is the main cause of clinical failure for veneered zirconia-based restorations ( Pjetursson, Sailer *et al.* 2015). Given the complexities of veneered zirconia-based restorations, clinicians and researchers have explored a variety of intraoral repair techniques to treat the chipping and protect the intact structures. Under appropriate conditions, intraoral repair acts as a potential therapy option. Dental professionals now have a great deal of interest in the scientific basis, therapeutic methods, and clinical effectiveness of the intraoral repair of cracked veneered zirconia-based restorations. Reviewing the intraoral repair of veneered zirconia-based restorations would improve practitioners' understanding of the advised procedures when zirconia repair is required ( Agingu, Zhang *et al.* 2018).

Understanding the complexities of porcelain fractures is the first step. Porcelain fractures can be static, cohesive, or adhesive. The material used for framework fabrication determines the repair procedure ( Aslam, Hassan *et al.* 2018). Regardless of their high 5-year survival rate of 90.4% (95% CI: 84.8–94.0%), porcelain-veneered zirconia FDPs can eventually crack and get chipped off. This is fundamentally due to the loss of adhesion between zirconia and veneering ceramics ( Pjetursson, Sailer *et al.* 2015). A variety of factors can cause the fracture of veneered porcelain on metal-ceramic crowns, which include lack of sufficient porcelain framework support, intra-ceramic flaws, or para-functional occlusion ( Haselton, Diaz-Arnold *et al.* 2001). Porcelain fracture can manifest clinically with no metal substrate exposure, modest metal substrate exposure, or significant metal exposure. Although fabricating a new crown seems desirable, it is not always possible. In such cases, the option of intraoral restoration is deemed of immense importance ( Haselton, Diaz-Arnold *et al.* 2001).

Before the build-up of the veneer layer, a succession of thermal sintering cycles and accompanying cooling processes are used to prepare crowns and FPDs. The matching of thermal expansion between the porcelain and the underlying framework, whether metal or ceramic, is crucial to avoid breaking following the firing. Delamination of the porcelain can occur when the coefficient of thermal expansion (CTE) of the framework is significantly higher than that of the porcelain ( Swain 2009). Other researchers believe that there should be a similarity in the CTE of both veneering porcelain and substructure to eliminate any stresses in the layered porcelain ( Aboushelib, Kleverlaan *et al.* 2006, Swain 2009). Fracture is a potential risk for veneered ceramics with such residual stress. A tensile stress zone can form below and at the edge of the contact surface of the veneer layer resulting from the combination of compressive residual stress and local stress brought on by mastication ( Taskonak, Mecholsky *et al.* 2005). Several elements influence the development of thermally induced residual stress and contribute to porcelain chipping which includes the veneer's overall thickness, cooling rate, and shape. Guazzato *et al.* (2010) found that rapid cooling and increased porcelain veneer thickness increase the risk of crack occurrence ( Guazzato, Walton *et al.* 2010). Zirconia may also crack due to the volume expansion (3–4%) occurring during the thermal tempering at high temperatures, which is done to facilitate tetragonal to monoclinic transition ( Piconi and Maccauro 1999).

Meeting the requirements for repairing a failed restoration is the next essential step. The establishment of a permanent bond between the previous restoration and the new repair material is the key to a successful repair. Therefore, choosing the right adhesive resin and restorative substance, as well as adequate surface conditioning of the substrate, is important. To increase bond strength, the surface is roughened using a variety of surface preparation processes ( Duzyol, Sagsoz *et al.* 2016). Acid etching using hydrofluoric acid (HF) is an optimal approach for roughening feldspar ceramic surfaces for resin composite bonding. In comparison with HF treatment alone, the introduction of a silane coupling agent after HF results in stronger resistant bonds. Further, newer surface modifications, such as surface abrading airborne particles, have been developed with advancements in adhesive technology. The initial step in this approach is air abrasion using aluminum oxide particles. Increased resin composite-porcelain bond strengths are reportedly achieved by sandblasting the porcelain's surface with aluminum oxide particles (35–250 μm) ( Duzyol, Sagsoz *et al.* 2016). After air abrasion, chemical adhesion can be generated using specialized primers that interact with a material's surface. To chemically bind the inorganic filler particles to the resin matrix, the most commonly used primer is a silane coupling agent, which is also employed in the construction of composites ( Loomans and Özcan 2016).

The wettability of the composite to be utilized as a repair material is increased by applying intermediate adhesive resin to the silanized surface. It is often recommended, but not required, to mix identical composite materials when doing composite-composite repairs due to the variable effects of different substrate materials ( Baur and Ilie 2013). Usage of adhesive resin, silanization, and HF acid etching, adhesion to glassy matrix ceramics has been thoroughly established. Optimal surface conditioning of indirect composite restorations can be achieved by using airborne particle abrasion followed by an adhesive resin with a silane coupling agent ( Loomans and Özcan 2016).

In the current review, HF etching in combination with laser treatment or silane and traditional adhesive was found to strengthen the link between porcelain and composite resin ( Hakimaneh, Shayegh *et al.* 2020, Benli, Kilic *et al.* 2022, Kiomarsi, Jarrah *et al.* 2022). However, durable TBS was observed when surface treatment with air abrasion or roughening the zirconia surface with a silicon carbide bur was offered for the repair of zirconia ceramic after chipping of its veneers ( Libecki, Elsayed *et al.* 2017).

## Conclusion

With the limitations of this narrative review, we can conclude that intraoral repair of ceramic fractures offers esthetics and comfort to patients when a restoration cannot be removed or changed. The current review indicated that the best technique to fix chipping in a complex made of zirconia core and veneering ceramic is to treat the veneering ceramic with HF before covering the zirconia core ceramic with silica. Both ceramic substrates must then be covered with adhesive resin and silane. The intraoral repair utilizing resin-based composite materials has significant advantages since it retains most of the restoration, prevents needless loss of healthy tooth structure, and is a quick and simple, reasonably priced procedure with no need for repeated sessions. The skills of the clinicians in using these tools and techniques as well as adherence to prescribed procedures and protocols are crucial to the effectiveness of these treatments.

## Data Availability

No data are associated with this article.
