# Supplementary material for: Intraoral repair of fractured ceramics: a literature review
Source: F1000Res. 2022 Nov 8;11:1275. [Version 1] doi: 10.12688/f1000research.126547.1 (PMC11615432; doi:10.12688/f1000research.126547.1)
Supplement: Peer review history

# Reviewer report: Reviewer response for version 1

**DOI:** 10.5256/f1000research.138970.r344694

**Published:** 2025-01-07

**Authors:** Punit Fulzele

**Affiliations:** 1 SPDC, Datta Meghe Institute of Higher Education & Research, Wardha, Maharashtra, India

This review addresses the intraoral repair of fractured dental ceramics, a common and clinically significant issue in dental practice. The authors conducted a non-structured PubMed search for articles published in English between 2017 and 2022, identifying 21 relevant studies. This review considers various techniques and ceramic types to provide a comprehensive overview of modern repair strategies for ceramic fractures and current repair protocols for different fracture types and core materials. It is a valuable resource for research and clinical practice due to its practical relevance and accessibility. Although the clinical studies were limited to 50 patients, the conclusions must be interpreted cautiously. There is an inconsistency in the reported timeframe for the included studies. Two critical aspects have not been discussed: the age of the restoration and the impact it has on the repair prognosis-surface pretreatment with plasma for improved bond stability.  The review's conclusions, while logical, are not fully supported by the included articles. There is, however, a well-structured summary of existing practices and recommendations for managing intraoral ceramic repairs. Despite some limitations, it offers dental professionals a valuable resource for understanding and implementing current repair strategies. Additional clinical data and the additional aspects mentioned could be included in future reviews to give a more comprehensive topic analysis.

Is the review written in accessible language?

Yes

Are all factual statements correct and adequately supported by citations?

Yes

Are the conclusions drawn appropriate in the context of the current research literature?

Partly

Is the topic of the review discussed comprehensively in the context of the current literature?

Yes

Reviewer Expertise:

CADCAM, Digital technologies, Dentistry.

I confirm that I have read this submission and believe that I have an appropriate level of expertise to confirm that it is of an acceptable scientific standard, however I have significant reservations, as outlined above.

# Reviewer report: Reviewer response for version 1

**DOI:** 10.5256/f1000research.138970.r344693

**Published:** 2025-01-02

**Authors:** Rafael Delgado-Ruiz

**Affiliations:** 1 Prosthodontics and Digital Technology, Stony Brook University, School of Dental Medicine, Stony Brook, New York, USA; 2 State University of New York at Stony Brook, Stony Brook, USA

The authors conducted a review of techniques for the intraoral repair of fractured dental ceramics, addressing a relevant and clinically significant topic for the journal's audience. Fractured ceramics represent a common complication in dental practice, often requiring effective clinical intervention and repair.

 The authors performed a non-structured search on PubMed for articles published in English between 2017 and 2022 related to intraoral ceramic repair, identifying 21 articles. Of these, 17 were in-vitro studies, 3 were case reports, and 1 was a prospective clinical study. However, the clinical data is limited, as the case reports and the prospective study together account for only 50 patients. Given this lack of robust clinical evidence, the conclusions of the manuscript should be interpreted with caution. 

 In addition, the second part of the conclusions " *The intraoral repair utilizing resin-based composite materials has significant advantages since it retains most* *of the restoration, prevents needless loss of healthy tooth structure, and is a quick and simple, reasonably priced procedure* *with no need for repeated sessions. The skills of the clinicians in using these tools and techniques as well as adherence to* *prescribed procedures and protocols are crucial to the effectiveness of these treatments." although logical, is not supported by the articles included in this review.*

Is the review written in accessible language?

Yes

Are all factual statements correct and adequately supported by citations?

Yes

Are the conclusions drawn appropriate in the context of the current research literature?

Partly

Is the topic of the review discussed comprehensively in the context of the current literature?

Yes

Reviewer Expertise:

Prosthodontics, CAD-CAM, Digital technologies,  Optical Scanners, Dental Implants, Thermography

I confirm that I have read this submission and believe that I have an appropriate level of expertise to confirm that it is of an acceptable scientific standard, however I have significant reservations, as outlined above.

# Reviewer report: Reviewer response for version 1

**DOI:** 10.5256/f1000research.138970.r344687

**Published:** 2024-12-03

**Authors:** Philipp-Cornelius Pott

**Affiliations:** 1 Hannover Medical School, Hannover, Germany

The authors have addressed a clinically highly relevant topic. The summarized literature is well suited to provide an overview of modern strategies for the repair of ceramic fractures. Particularly noteworthy is the fact that the authors consider not only various repair techniques but also various ceramics in a differentiated way. This nicely highlights the fact that not every repair strategy can be applied equally to all materials or material combinations. I also particularly like the fact that the authors address possible causes of damage to restorations at the beginning of the discussion.

 In the methodology section, chapter 2.1, it is stated that studies published between January 2017 and August 2022 have been included. However, a different time period (2016 to 2022) is given in the first chapter of the discussion. This is probably just a typo, which should be easy to correct.

 Nevertheless, a fairly extensive presentation of intraoral ceramic repair has already been made here, but unfortunately I miss two important aspects that should be added to the discussion:

 1. Age of the restoration to be repaired.

 Unfortunately, silicate ceramics absorb moisture over time. This moisture absorption cannot be reversed intraorally. Accordingly, this reduces the prognosis for a secure, stable bond. In zirconium dioxide ceramics, so-called low-temperature degradation occurs over time under the conditions prevailing intraorally (Borchers et al Ref 1; Kohorst et al. Ref 2). This also reduces the long-term prognosis of a restoration that may need to be repaired.

 2. Surface pretreatment with plasma.

 The application of cold argon plasma to ceramic surfaces, for example, increases hydrophilicity and thus improves wettability with adhesives, thereby increasing the long-term stability of the ceramic-composite bonds (Pott et al. ref 3). Corresponding application devices are also available for dentistry.

Is the review written in accessible language?

Yes

Are all factual statements correct and adequately supported by citations?

Yes

Are the conclusions drawn appropriate in the context of the current research literature?

Yes

Is the topic of the review discussed comprehensively in the context of the current literature?

Yes

Reviewer Expertise:

dentistry, adhesive dentistry, adhesion to ceramic materials and metals, prosthodontics, all-ceramics

I confirm that I have read this submission and believe that I have an appropriate level of expertise to confirm that it is of an acceptable scientific standard.

## References

[1] : Influence of hydrothermal and mechanical conditions on the strength of zirconia. *Acta Biomater* .2010;6(12) : 10.1016/j.actbio.2010.07.025 4547-52 10.1016/j.actbio.2010.07.025 20656076

[2] : Low-temperature degradation of different zirconia ceramics for dental applications. *Acta Biomater* .2012;8(3) : 10.1016/j.actbio.2011.11.016 1213-20 10.1016/j.actbio.2011.11.016 22138551

[3] : Influence of nonthermal argon plasma on the shear bond strength between zirconia and different adhesives and luting composites after artificial aging. *J Adv Prosthodont* .2018;10(4) : 10.4047/jap.2018.10.4.308 308-314 10.4047/jap.2018.10.4.308 30140398 PMC6104498

# Reviewer report: Reviewer response for version 1

**DOI:** 10.5256/f1000research.138970.r155374

**Published:** 2022-11-28

**Authors:** Lomaya Ghanem

**Affiliations:** 1 Fixed Prosthodontics Department, Faculty of Dentistry, Misr International University, Cairo, Egypt

Intraoral repair of fractured veneering ceramic is a very relevant, economic and conservative clinical approach. The review herein clearly exhibits the fracture types, their conforming repair protocol in relevance to the restoration core material (metal or ceramic), highlighting procedures and materials used. The herein review is supported with literature consistent with current research. Language, context, and drawn conclusions: clear

 It furnishes a practical source for research, and provides an accessible overview and understanding of the topic, as well as recommendations for management of intraoral repair using the existing practices.

Is the review written in accessible language?

Yes

Are all factual statements correct and adequately supported by citations?

Yes

Are the conclusions drawn appropriate in the context of the current research literature?

Yes

Is the topic of the review discussed comprehensively in the context of the current literature?

Yes

Reviewer Expertise:

Fixed Prosthdodontics, ceramics, pressing, bonding, CADCAM.

I confirm that I have read this submission and believe that I have an appropriate level of expertise to confirm that it is of an acceptable scientific standard.
